# Rewarding the quantity of peer review could harm biomedical research

**DOI:** 10.11613/BM.2019.020201

**Published:** 2019-04-15

**Authors:** Aceil Al-Khatib, Jaime A. Teixeira da Silva

**Affiliations:** 1 Faculty of Dentistry, Jordan University of Science and Technology, Irbid, Jordan; 2 P. O. Box 7, Miki-cho post office, Ikenobe 3011-2, Kagawa-ken, Japan

**Keywords:** autonomy, biomedical research, ethics in publishing, responsible peer review, scientific misconduct

## Abstract

Voluntary peer review is generally provided by researchers as a duty or service to their disciplines. They commit their expertise, knowledge and time freely without expecting rewards or compensation. Peer review can be perceived as a reciprocal mission that aims to safeguard the quality of publications by helping authors improve their manuscripts. While voluntary peer review adds value to research, rewarding the quantity or the volume of peer review is likely to lure academics into providing poor quality peer review. Consequently, the quantity of peer review may increase, but at the expense of quality, which may lead to unintended consequences and might negatively affect the quality of biomedical publications. This paper aims to present evidence that while voluntary peer review may aid researchers, pressurized peer review may create a perverse incentive that negatively affects the integrity of the biomedical research record. We closely examine one of the proposed models for rewarding peer review based on the quantity of peer review reports. This article also argues that peer review should remain a voluntary mission, and should not be prompted by the need to attain tenure or promotion.

## Introduction

**Figure 1:**
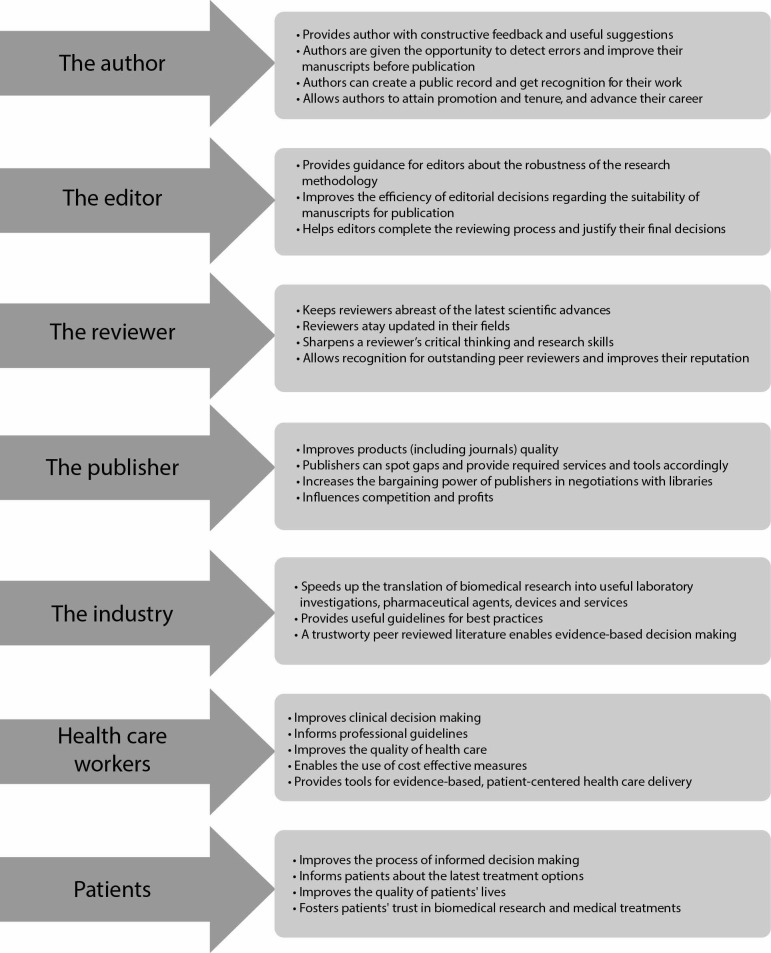
Examples of how each stakeholder benefits from quality peer review

Trustworthy peer reviewed publications enable clinical practice guidelines to be developed and updated (*1*). Such guidelines are central to services that biomedical researchers provide. If timely and responsible peer review is implemented, it will speed up the translation of biomedical or health research into useful policy and practice (*2*). It is therefore important for active researchers to support the peer review process, each by contributing to their field of expertise, because safeguarding of the literature’s appropriate scientific standards and the sustainability of scientific research depend on the contribution of expert scientists to this process (*3*). Peer review does not operate within a vacuum. Authors, editors, reviewers, publishers, industries, policy makers, healthcare workers and patients benefit from peer review (Figure 1). Even after an article is published, critical analyses or post-publication peer review have as much or more value than pre-publication peer review (*4*). However, for peer review to work effectively, the contribution of all members needs to be fair and balanced. Recent calls for institutions to track the peer review services that researchers provide, and to use a peer review metric in assessing and promoting academics, raise concerns because researchers may be placed under pressure to provide peer review in order to advance their careers (*5*). This article aims to assess whether peer review should be a voluntary or mandatory mission, and discusses potential drawbacks of pressurizing authors into providing peer review.

## Is peer review a voluntary or a mandatory mission?

There are many reasons why biomedical researchers contribute to peer review. The benefits of peer reviewing manuscripts are numerous not only to academics, but also to academics’ professions and disciplines. Even though peer reviewers benefit from keeping abreast with research developments in their fields, and from improving the quality and integrity of research publications, these benefits are incomparable to the profits that some publishers make from selling the products that result from the efforts of peer reviewers (*6*). Although some journals provide financial incentives to peer reviewers, the bulk of peer review is performed by a voluntary and unpaid service by researchers, clinicians, experts and academics (*7*). Analysing the online information displayed on Publons website demonstrates that over 575,000 researchers have contributed more than 1.5 million peer review reports (*8*, *9*). Based on the approach of the *American Economic Review*, which pays 100 US dollars for timely peer review reports, it is not surprising to assume that the monetary value that was not charged (by peers) to publishers to perform peer review is in the range of at least 150 million US dollars, had researchers been paid 100 US dollars *per* peer review report (*10*). In other words, massive profits publishers currently make would not be made without the contribution of volunteering peer reviewers. The result of perceived injustice by some may have contributed to the shortage of peer reviewers, so that finding suitable reviewers with sufficient experience and skills has become a challenging and time-consuming task. Interestingly, Heinemann the editor of *Journal of Diabetes Science and Technology*, a SAGE journal, raised the issue of whether a reviewer was an “endangered species” (*11*). One solution to finding peer reviewers was to establish a database of reviewers, and to consider options that would allow the recognition of peer review to attract more peer reviewers to the process of validating scientific publications (*12*-*14*). One has to wonder why this had not occurred to all stakeholders of the publishing process prior to 2014-2016. The idea of providing meaningful recognition for peer review activity is encouraged and is long overdue, but it can be argued that pressuring or inducing academics to provide peer review services is likely to result in questionable practices, practices that are likely to impair the quality of published biomedical research because if the process is forced and is not natural, it could lead to the creation of perverse incentives (*15*).

## Potential drawbacks to pressured or induced peer review

Herman remarked that “the moral status of an action is connected to an agent’s judgment and choice” (*16*). Thus, if academics are forced or pressurized to peer review to receive extra points and pad their portfolios with peer review reports, it will lead to an increase in the number of peer reviewers, but will not necessarily lead to improving the quality of peer review. This outcome is likely, as demonstrated by Fiala and Willett, who showed that the “quality” of publications, as measured by the Clarivate Analytics^TM^ journal impact factor (JIF), was not accompanied by a substantial increase in the rate of publications (*17*). When they averaged the JIF, they found that the mean JIF dropped from 1.455 for 1989-2000 and to 1.302 for 2001-2014 (*17*).

Another disadvantage of pressuring academics to perform peer review may lead to increased competition between academics so that the number of academics chasing peer review in order to pad their *curriculum vitae* is likely to increase. If such a demand is not provided by reputable journals, some academics, under pressure of their job requirement, will have no option but to provide services to an increasing market of unscholarly or “predatory” journals or publishers in order to fulfil this enforced requirement. By providing pressured peer review to “predatory” journals, reviewers would in essence provide a mask of legitimate peer review to journals with questionable publishing practices. Needless to say that such journals, under increasing pressure, need to provide “any, even if meaningless” peer review to improve their image and justify their article processing charges, although it could then be argued that if they provide legitimate peer review, then they are not “predatory” journals. The issue is a grey area since fake peer reviews plague both “predatory” journals as well as indexed journals that are traditionally considered to be safe.

## Juxtaposing the current model of “rewarding peer review” with “publish or perish”

The Publons model, which offers academics recognition for their activity in providing peer review, and rewards them for the number of peer review reports, should not ignore the fact that peer review has been and should remain a voluntary task (*18*, *19*). If this model is misused, it may pressurize authors. In other words, if peer review becomes a mandatory requirement for tenure, the threat of job loss is likely to jeopardize academics’ (*i.e*., peer reviewers’) free will, and add a layer of pressure. Arguably, pressurized peer reviewers are unlikely to improve the quality of peer review. Early manifestations of the “publish or perish” mandate made publications a mandatory requirement for tenure in many countries (*20*). There are signs that the Publons model is using marketing strategies that can be misused and could expose academics to the risks of coercion (*21*). The Publons model was used by three journals that appointed a dog to their editorial boards in a sting by Professor Mike Daube, a public health expert in Perth, Australia: *EC Pulmonology and Respiratory Medicine*, *Journal of Community Medicine and Public Health Care* and *Journal of Tobacco Stimulated Diseases* (*22*-*25*). Pressure to peer review may lead to consequences that are similar to those witnessed in the publish or perish culture and its association with research misconduct such as paying fake peer reviewers or exploiting non-expert junior researchers who are less likely to provide critical peer review or carefully scrutinize manuscripts before they are published (*26*).

The pressure to peer review is similar to the pressure to publish, and the latter has led to publication bias and compromised the objectivity and integrity of research (*27*). Although these strategies discussed above are used in good faith to encourage academics to contribute to peer review, they include messages that could lead to implementing a criterion of peer review by promotion and tenure committees, or to adding another perverse incentive (*28*). Furthermore, readers are urged to consider who may be targeted by a recommendation to include a verified peer review record when seeking a visa or Green Card in the United States of America (*29*). A computational materials scientist at the University of Florida, who had secured his Green Card for the United States of America, shared with Publons how he used statistics on his Publons Verifiable Review Record, offered tips on how to use peer review to boost Green Card applications, and advised researchers to make their peer review public.

Publons awards the number of papers that have been peer reviewed by displaying the number of verified reviews, the number of reviews in the last six months, and the number of verified editors record for each reviewer. Thus, quantifying peer review activity may be erroneous and unfair to experts in a niche field because they are likely to receive very few invitations to peer review by virtue of the fact that very few articles are published in their narrow field. Conversely, a researcher who is one of few experts in a rather popular field may get more review invitations because there are not many reviewers available in that field.

It should be cautioned that the competition between peer reviewers striving to boost their Publons profiles with more and more reports might increase peer review fraud, or dishonesty, or at least compromise the quality of peer review and its products. Altman remarked that the pressure to publish created a temptation to behave dishonestly when “all too often the main reason for a piece of research seems to be to lengthen a researcher’s curriculum vitae” (*30*). With Altman’s statement in mind and given the gradual increasing transition to open access and the inevitable explosion of journals, can biomedical researchers bear the consequences of being further pressurized into showing a lengthy peer review record as evidence of scholarly contribution or productivity?

## Conclusion

Pressuring or inducing academics into creating a list of peer review reports raises serious questions about the quality of such peer review and the value that such an action adds to biomedical research. To encourage researchers to participate in efficient peer review, we recommend eliminating sources of pressure, preventing violations of researchers’ rights, protecting vulnerable early career researchers from being pressured or induced into providing non-expert peer review, and encouraging and rewarding quality voluntary peer review (*31*, *32*). Rewarding reviewers who provide quality peer review can be achieved by paying peer reviewers for their time and expertise. The adoption of an open peer-review system wherein experts engage in validating and grading peer review according to specific guidelines for each scientific field is feasible (*33*). Finally, promoting a sense of shared responsibility, and encouraging experts to provide post publication peer review, is likely to weed out poor reviewers, and instil a culture of responsible peer review of biomedical research.
